# Fluorinated graphene-modified biodentine: an in vitro study on its ion release, cell growth, differentiation potential, and compressive strength

**DOI:** 10.1186/s12903-025-06947-7

**Published:** 2025-10-31

**Authors:** Fagr Hassan Elmergawy, Iman Ahmed Fathy, Zeinab Mahmoud Abulwafa, Ghada Ahmed Elzayat, Heba Mahmoud Seif

**Affiliations:** 1 Lecturer of Dental Biomaterials, Faculty of Dentistry, October University for Modern Sciences and Arts (MSA), 26th July Mehwar road intersection with Wahat road, 6th October City, Egypt; 2 Associate professor of Oral Biology, Faculty of Dentistry, Ain Shams University, El-Khalifa El-Mamoun Street, Abbasya, Cairo, Egypt; 3 Lecturer of Oral Biology, Faculty of Dentistry, Al Ryada University, Al-Sadat City, Egypt; 4 Lecturer of Conservative dentistry, Faculty of Dentistry, Egyptian Russian University, Cairo, Egypt; 5 Lecturer of Dental Biomaterials, Faculty of Dentistry, Fayoum University, Fayoum, Egypt

**Keywords:** Bioactivity, Biodentine, Compressive strength, Fluorinated graphene, Odontogenic differentiation

## Abstract

**Objectives:**

This study evaluated Biodentine (BD) after modification with 2 wt% fluorinated graphene (FG).

**Methods:**

FG was prepared using the modified Hummers’ method, where sulfuric and phosphoric acids were added to fluorinated graphite and potassium permanganate. The mixture was heated, sifted, filtered, and centrifuged to obtain FG powder. Characterization was performed using XRD, FTIR, TEM, and SEM/EDX. PH was evaluated, And Ca And F ion release were assessed by inductively coupled plasma spectroscopy and ion chromatography, at days 1,14, And 28. Cell viability was performed using the MTT Assay on pulp stem cells, while ALP assay was evaluated by a spectrophotometer. Compressive strength was evaluated by a universal testing machine. Statistical analysis was performed on the data (*p* ≤ 0.05).

**Results:**

Graphene and C-F bonds of FG were confirmed in XRD and FTIR, while nanosheets were detected in TEM. SEM/EDX showed more surface roughness in modified BD-FG. pH And Ca ion release results showed significantly higher values at day 1 for modified BD-FG, with significantly higher cumulative Ca ion release. Cell viability results showed no significant difference between modified And unmodified Biodentine at days 1 And 7; however, modified BD-FG showed significantly lower values at day 3. No significant difference was observed between the two groups in ALP, while the BD-FG group showed significantly higher compressive strength.

**Conclusion:**

Incorporating 2 wt% FG into BD increases ion release, hydroxyapatite formation, and mechanical properties without compromising cell viability and differentiation.

**Clinical relevance:**

The addition of FG enhanced the bioactivity of Biodentine and improved its strength without showing cytotoxicity, making it a promising approach that needs further study.

**Supplementary Information:**

The online version contains supplementary material available at 10.1186/s12903-025-06947-7.

## Introduction

Mineral trioxide aggregate (MTA) was first introduced in 1993 and is considered the first bioceramic material applied in endodontics that was developed based on Portland cement [1]. MTA exhibited good biocompatibility and sealing ability, in addition to apatite formation with a higher rate of clinical success. MTA can form of thicker and less porous dentin bridge with fewer signs of inflammation. However, it shows some inherent limitations, such as its prolonged setting time, difficult handling characteristics, possibility of tooth discoloration, as well as its high cost [1, 2].

Many modified MTA products were introduced in the market to overcome the shortcomings of traditional MTA while retaining its original excellent performance. One of these products is Biodentine (Septodont, Saint-Maur-des-Fosses Cedex, France), which is considered a second-generation calcium silicate-based material. It was introduced to the market in 2011 as a bioactive dentin substitute due to its ability to penetrate and crystallize into open dentinal tubules, provide interlocking with dentin, and increase its mechanical properties [3]. One of the advantages of Biodentine over traditional MTA is its shorter setting time, higher viscosity, easier application and handling characteristics, and less tendency for tooth discoloration. Moreover, it shows very promising bioactivity, which comes mainly from being formed of tri-calcium silicate and calcium carbonate matrix along with zirconium oxide and iron oxide. Biodentine has been extensively used as a pulp capping material, retrograde filling, perforation repair, treatment of immature necrotic teeth, pulpotomy, and apexification [4]. Despite the desirable properties of Biodentine, many studies have been conducted to incorporate different nanomaterials into Biodentine to improve its physical, biological, and mechanical properties [5–8].

Graphene oxide (GO) is a biocompatible material derivative of graphene that can be safely incorporated into various biomedical applications. Graphene was discovered in 2004, and it is composed of a two-dimensional honeycomb lattice of carbon atoms. It has been extensively studied due to its high mechanical properties, chemical stability, low cytotoxicity, and antibacterial effects. Moreover, it was reported that graphene nanocomposites could promote cell adhesion and proliferation, facilitating wound healing, and acting as frameworks for regenerative medicine [9], as well as enhancing nucleation and crystallization of hydroxyapatite in biomimetic conditions [10, 11]. Moreover, GO exhibited a good strengthening effect when added to different dental biomaterials [12–14].

Nevertheless, the application of graphene in modern restorative dentistry is quite restricted owing to its dark color [15]. Fluorinated graphene (FG) is a fluorine-functionalized graphene that shares the same morphological pattern as graphene. It exhibits great potential for application in the dental field because of its bright white color, besides the unique properties of graphene [16]. Moreover, the potential of fluoride release from FG can favor tooth remineralization and prevent or treat early demineralized tooth lesions owing to the ability of fluoride to promote the formation of more stable, acid-resistant fluorapatite crystals [16]. FG also exhibits better dispersion than pure graphene or graphene oxide due to the presence of C-F bonds, causing the reduction of surface energy of the carbon sheets and thus allowing their proper distribution and decreasing their agglomeration [17]. It also shows high stability owing to the presence of covalent bonding between C-F with a high difference in electronegativity between both elements, which improves the local symmetry, electronic structure, and stability of FG [18].

It has been reported that the addition of FG nanofiller showed promising results when added to different dental materials, such as glass ionomer [18–21] and dental adhesives [22, 23]. But to the best of our knowledge, research regarding the incorporation of FG into Biodentine is still lacking.

Based on preliminary pilot experiments, the incorporation of FG into Biodentine demonstrated An improvement in mechanical properties, with the optimal effect observed at a concentration of 2wt.%. Beyond this concentration, no further improvement was noted. Hence, this study aimed to incorporate 2 wt% FG into Biodentine and evaluate its pH, Ca ion release, as well as investigate its effect on dental pulp stem cells regarding cell viability and alkaline phosphatase activity, besides evaluating its compressive strength in comparison to unmodified Biodentine.

The null hypothesis states that there would be no statistically significant difference between the unmodified Biodentine group and the FG-modified Biodentine group on the different tested properties.

## Materials and methods

All participants provided written informed consent, and the study protocol received approval from the Research Ethics Committee of the Faculty of Dentistry at Ain Shams University (FDASU-Rec IR022522), following the Helsinki Declaration. The study was conducted over a period of six months.

### Fabrication of fluorinated graphene (FG)

Fluorinated graphene powder was prepared following the modified Hummers’ method mentioned in a previous study [24]. Where, a mixture of concentrated sulfuric and phosphoric acid (H_2_SO_4_/H_3_PO_4_) in a ratio of 9:1 was added to a mixture of 3 g of fluorinated graphite flakes (Graphite Fluorinated Polymer; Sigma Aldrich, China) And 18g of potassium permanganate (KMnO_4_). The mixture was then heated to 50 °C And stirred for 12h using a magnetic stirrer. The reaction was then carefully poured onto ice for cooling. The resulting mixture was sifted through a metal sieve And then filtered through a polyester fiber. The filtrate was centrifuged, And the supernatant was decanted away. The remaining solid material was then washed in succession with 200 mL of water, 200 mL of 30% HCl, And 200 mL of ethanol for each wash. Then the mixture was sifted again, filtered, and centrifuged as described above to obtain white-colored FG powder. The obtained sample powder was dried and kept in a tightly sealed container till testing.

### Preparation of FG incorporated biodentine

Biodentine (BD group) (Septodont, Saint-Maur-des-Fosses Cedex, France) was prepared following the manufacturer’s instructions, where Biodentine Liquid was added to the BD containing capsule And mixed in An amalgamator for 30s at 4500 rpm to obtain a creamy consistency paste. To prepare FG-modified Biodentine (BD-FG group), 2 wt% FG powder (based on a pilot study) was added to BD powder. Since the Biodentine powder weight is 0.7gm, the weight of 2 wt% FG was calculated to be 0.014gm. FG powder (0.014 g) was added to the BD capsule, followed by immediate mixing in An amalgamator for 30s to ensure its dispersion within the BD powder. Afterwards, liquid was added and mixed following the manufacturer’s instructions as mentioned above. The liquid-to-powder ratio was maintained throughout specimens’ preparation by carefully weighted the Biodentine powder and measuring the volume of its liquid, and keeping this ratio constant across all the specimens.

### Characterization of FG and BD-FG

Crystalline phase identification of FG powder was characterized by X-ray diffraction analysis (XRD) using X-ray diffractometer (PXRD-6000 SCHIMADZU Japan) operated with a current of 30 mA And voltage of 40kV with CuKα radiation (λ = 1.54056A^o Å^), And over 2Ө Angle range of 5^o^ to 80^o^. Functional group analysis was assessed by Fourier Transform Infrared Spectra (FTIR) (IRAffinity-1 S, SHIMADZU, Japan) with a wavelength range from 4000 to 400 cm^−1^. Morphological analysis of FG powder was evaluated by transmission electron microscope (TEM) (JEM-2100, JEOL.USA) operated at An accelerating voltage of 200kV and was coupled with a CCD camera (Erlangshen ES500W Gatan Inc).

Phase identification and functional group analysis of Biodentine before and after modification with FG was also performed by XRD and FTIR respectively, using the same machines and parameters as mentioned above. While their morphological analysis was performed by scanning electron microscope (SEM) (Quanta FEG-250, FEI USA) coupled with EDX (Energy dispersive x-ray spectroscopy) elemental composition analyzer (EDAX-AMETEK, Inc., Netherlands) with An accelerating voltage of 20kV for elemental analysis. Elemental mapping of fluoride (F) was used to evaluate the dispersion of FG within the BD-FG group.

### Ph behavior and ion release profile 

Cement discs from each group (*n* = 10) were prepared using custom-made split Teflon molds of 10 mm diameter And 2mm height. The cements were mixed And packed in the molds between two glass slides covered with celluloid strips. The specimens were incubated at 37 ◦C in 100% relative humidity in an incubator (Titanox A3-213-400, Italy) for 24 h to ensure complete setting. Discs were then submerged in 10 mL of Hanks’ Balanced Salt Solution (HBSS) (Biowest, Nuaillè, France) in a sealed 50 mL sterile polypropylene tube And kept in An incubator at 37°C.

The pH was measured using a pH meter (HI98103, Hanna Instruments Inc., USA). Triplicate readings were recorded for each sample, and their average was calculated. Ca ion release was analyzed using an inductively coupled plasma mass spectroscopy (ICP/MS) (Agilent 7700, Agilent Technologies, Germany). Fluoride ion release from the modified BD-FG was evaluated using ion chromatography (Dionex ICS 6000, Thermo Scientific, USA). Evaluation of pH, calcium ion, And fluoride ion release was assessed after 1, 14, And 28 days.

### In vitro apatite formation ability

The cement discs were collected after 1, 14, And 28 days, and their surfaces were tested without any modification or coating using an environmental scanning electron microscope (ESEM) at magnification of 2000 X, operated at An accelerating voltage of 20KV, and attached to an Energy Dispersive X-Ray Spectroscopy (EDX Unit) (Quattro S, Thermofisher, USA). Elemental analysis using EDX was utilized to estimate the surface calcium-to-phosphorus (Ca/P) atomic ratios. The discs were returned to a new 10 mL of HBSS after 1 And 14 days and kept in the incubator until the following interval.

### In vitro assay

#### Isolation of dental pulp stem cells (DPSCS)

DPSCs were isolated from extracted permanent premolars And third molars, which were extracted for orthodontic purposes from patients between 18 And 25 years of age. The extracted teeth were rinsed and transferred to a washing solution containing Dulbecco’s phosphate-buffered saline (DPBS; Invitrogen™, Carlsbad, CA, USA) and an antibiotic mix (100 units/mL penicillin (Sigma), And 100µg/mL streptomycin (Gibco™ 250 µg/mL). A 0.5–1.0 mm deep groove was cut around each tooth using a sterile low-speed disc, and the teeth were then split with a chisel along the groove to expose the pulp. The extracted pulp tissues from all teeth were combined and cut into small pieces using a surgical scalpel, then digested in collagenase type I (3 mg/mL, Invitrogen™) in two 30-minute cycles at 37˚C. The resulting cell suspensions were cultured in a DPSC medium containing D-modified Eagle’s medium (GIBCO™ 1 g/l glucose, pyruvate +), 10% fetal bovine serum (Hyclone™), 100 IU/mL penicillin (Sigma™), And 100µg/mL streptomycin (Invitrogen™). The DPSCs were cultured at 37˚C with 5% CO2, with fresh medium replaced every two days, And cells were harvested at 80% confluence for optimal results. Cells were processed inside the Laminar flow cabinet (Thermo Scientific safety Cabinet MSc Advantage 1.2, class II HEPA H filter).

#### Cell viability test

MTT assay was conducted to evaluate the effect of both tested materials on cell viability. Discs of Biodentine And modified Biodentine were prepared, as mentioned above, And allowed to set in a 96-well plate for 24 h at 37 °C. Cells were then inoculated with seeding density of (1 × 10^4^ cells/well), where cells were allowed to settle on the discs of tested materials for the tested time intervals (1,14, And 28 days) And incubated at 37°C in a CO₂ incubator to form a complete monolayer sheet.

A working solution of a concentration of 5 mg/mL MTT solution (Elabsciences™ Cat. No PB180519) in phosphate-buffered saline (PBS) was prepared. 20 µL of the MTT working solution was added to each well of the 96-well plate And incubated for 2–4 h at 37 °C. 100 µL of DMSO (dimethyl sulfoxide) per well was then used to dissolve the purple formazan crystals in 10–15 min. Thermo Scientific, Multiscan Sky microplate spectrophotometer, And Skanlt Software 6.0.2 for Microplate Readers were used to measure the absorbance at 570 nm for viable cells And the background absorbance at 630nm. The absorbance values were used to calculate the percentage of cell viability compared to the control (untreated) cells using the following formula:

$$\begin{aligned}&\mathrm{Cell}\;\mathrm{Viability}\;(\%)\;=\\&\;(\mathrm{Absorbance}\;\mathrm{of}\;\mathrm{treated}\;\mathrm{cells}/\mathrm{Absorbance}\;\mathrm{of}\;\mathrm{control}\;\mathrm{cells})\;\\&\times100\end{aligned}$$

Cytotoxicity was classified according to ISO 10993-5 guidelines: non-cytotoxic (> 80% viability), slightly cytotoxic (60–80%), moderately cytotoxic (40–60%), and severely cytotoxic (< 40%).

#### Alkaline phosphatase (ALP) assay

ALP assay was used to evaluate the osteogenic differentiation of cells with the two tested materials. Cells were seeded on a 96-well plate with a density of 1 × 10^4^ cells/well. Cells were allowed to adhere And grow for 24h in osteogenic medium containing 10^−4^ M dexamethasone, 1 M β-glycerol-phosphate, And 10mg/mL ascorbic acid (all brought from STEM CELL Technologies, Canada) at 37 °C in a CO₂ incubator to reach optimal confluence. Afterwards, discs of Biodentine And modified Biodentine were prepared, allowed to set for 24h at 37 °C, And then distributed on the 96-well plate according to the study design. ALP assay reagents (MG Science And technology center, Cat. No MG 214001) were prepared by mixing 200 µL of assay buffer with 5 µL of Mg²⁺ solution And 2 µL of para-nitrophenyl phosphate (pNPP) substrate per well. Thermo Scientific, Multiscan sky microplate spectrophotometer, And Skanlt Software 6.0.2 for Microplate Readers were used to measure the absorbance at 405 nm for p-nitrophenol released, which in turn reflects the ALP activity in the cells And the background absorbance at 630nm. The absorbance values obtained were used to calculate the alkaline phosphatase activity, using the following formula:

$$\begin{aligned}\mathrm{ALP}\;\mathrm{Activity}\;&=\;(\mathrm A\;\mathrm{sample}-\;\mathrm A\;\mathrm{blank})\;\\&\mathrm x\;\mathrm{volume}\;\mathrm{in}\;\mathrm{mL}/\mathrm{time}\;\mathrm x\;\mathrm{cell}\;\mathrm{number}\end{aligned}$$

To account for potential variations in cell number, ALP activity was normalized to cell viability, which was concurrently assessed using the MTT assay. Normalized ALP values were expressed as ALP absorbance per unit of viable cell absorbance (ALP/MTT).

### Compressive strength testing

Cylindrical cement specimens from each group (*n* = 10) were prepared using custom-made split Teflon molds of 4 mm diameter And 6mm height. The cements were mixed And packed in the molds between two glass slides covered with celluloid strips. The specimens were incubated at 37 ◦C in fully saturated humidity for 24 h to ensure complete setting [14]. Compressive strength was evaluated using a universal testing machine (Instron 3365; Massachusetts, UK) with a load cell of 5 K And crosshead speed of 0.5 mm/min. Compressive strength in MPa was recorded from the peak of the recorded load divided by the specimen surface according to the following formula:

$$\mathrm{CS}\;=\;4\mathrm P\;/\;\uppi\mathrm{d}^2$$

Where P is the maximum loading in Newtons and d is the diameter of the specimen in mm.

### Statistical analysis

Means and standard deviation (SD) values for each tested group were explored for normality using Shapiro-Wilk’s test, which revealed a parametric distribution. Comparison between the unmodified and modified BD groups regarding cumulative Ca ion release, ALP activity, and compressive strength was analyzed by using an independent t-test. Two-way mixed model ANOVA was used to evaluate the interactions between different variables in pH and Ca ion release, while the correlation analysis between pH and Ca ion release was made by Spearman’s rank-order correlation coefficient. Comparison between the two tested groups with the control group in cell viability testing was analyzed by using One-Way ANOVA, followed by Tukey’s post hoc test. The significance level was set at *p* < 0.05 within all tests. Statistical Analysis was performed with R statistical Analysis software version 4.4.2 for Windows.

## Results

### Characterization of FG results

**Fig. 1:**
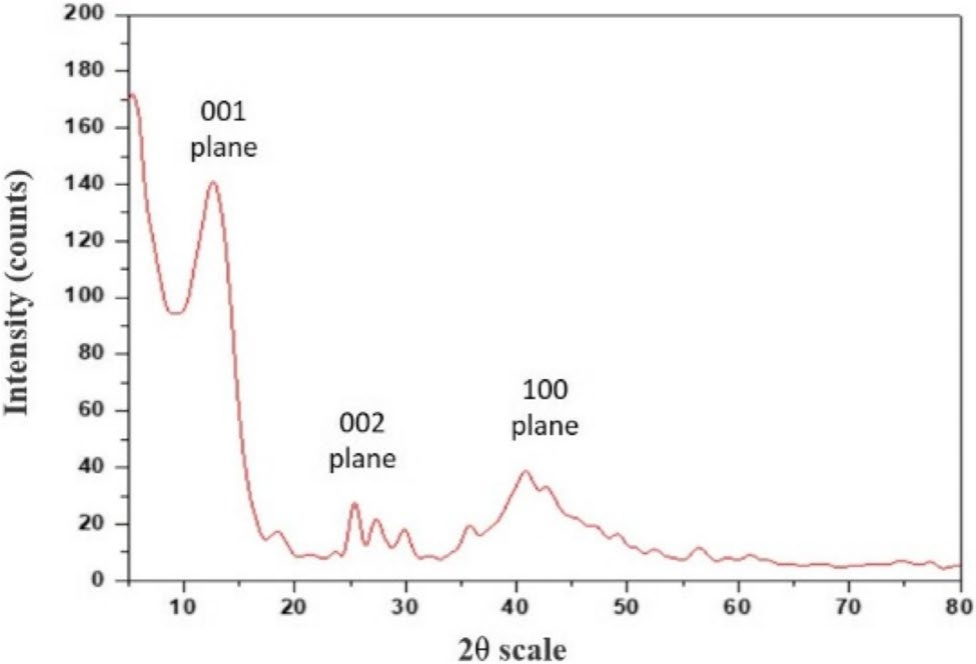
XRD pattern of FG powder

The XRD analysis of FG (Fig. 1) revealed characteristic peaks of graphene at 2θ = 13.78°, 25.51°, And 41.7° attributed to the diffraction of the crystal planes of (001), (002), and (100), respectively [25].

**Fig. 2:**
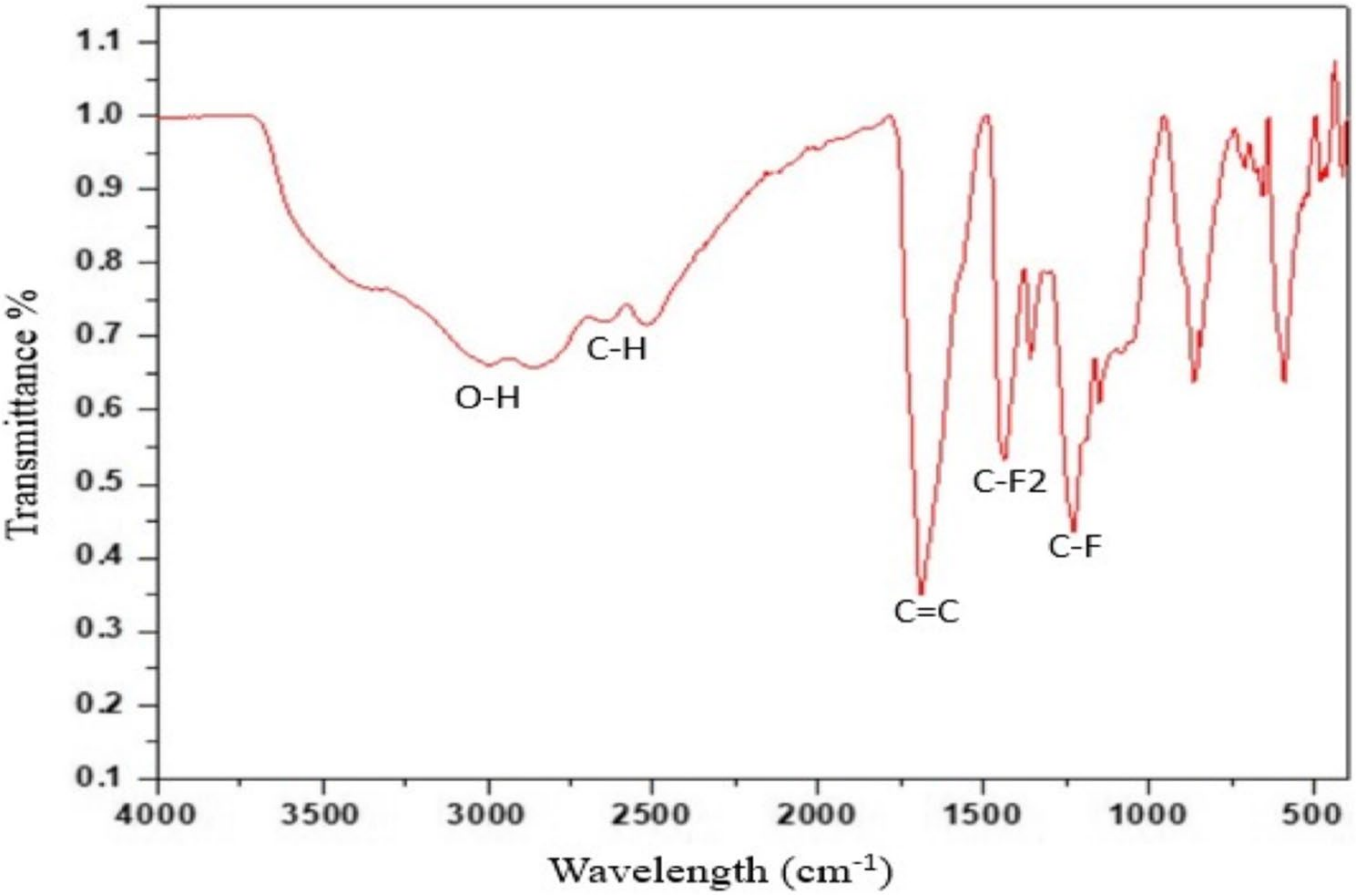
FTIR spectrum of FG powder

FTIR analysis of FG (Fig. 2) exhibited peaks corresponding to the stretching vibration of C-F bonds at about 1233 cm^−1^. Another peak was detected at 1450cm^−1^ corresponding to the C-F2 stretching vibration. A characteristic peak attributed to C = C bonds of graphene basal plane was observed at approximately 1690 cm^−1^. Also, the peak corresponding to the C-H bond was revealed at approximately 2600 cm^−1^. Stretching vibrations of OH groups were detected in the region of 3000 cm^−1^ [26, 27].

**Fig. 3:**
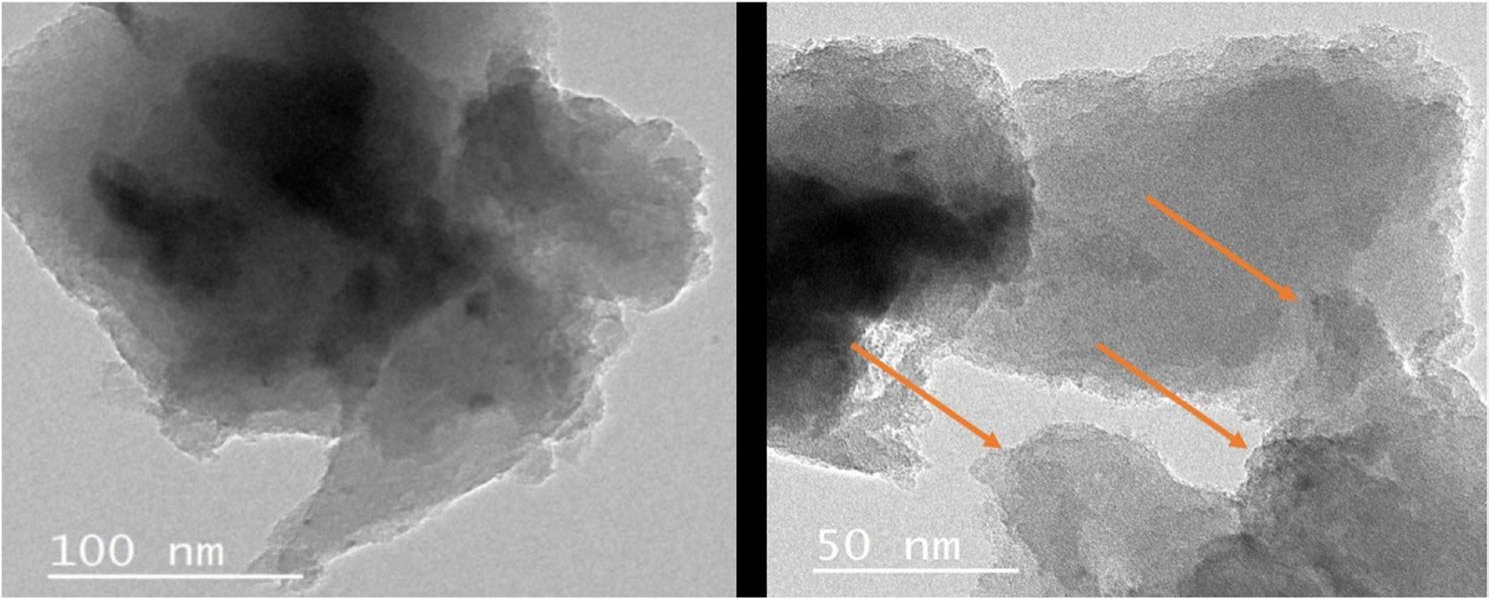
TEM images of FG at a scale bar of 100 nm And 50nm. Arrows denote the nanosheet structure

TEM image of FG (Fig. 3) showed FG nanosheets with lower opacity edges, indicating a high degree of exfoliation, and dark opaque areas in the middle, indicating stacking of the particles. The nanosheet lamellar structure of graphene can be detected (arrows).

### Chracterization of biodentine before and after modification with FG

**Fig. 4:**
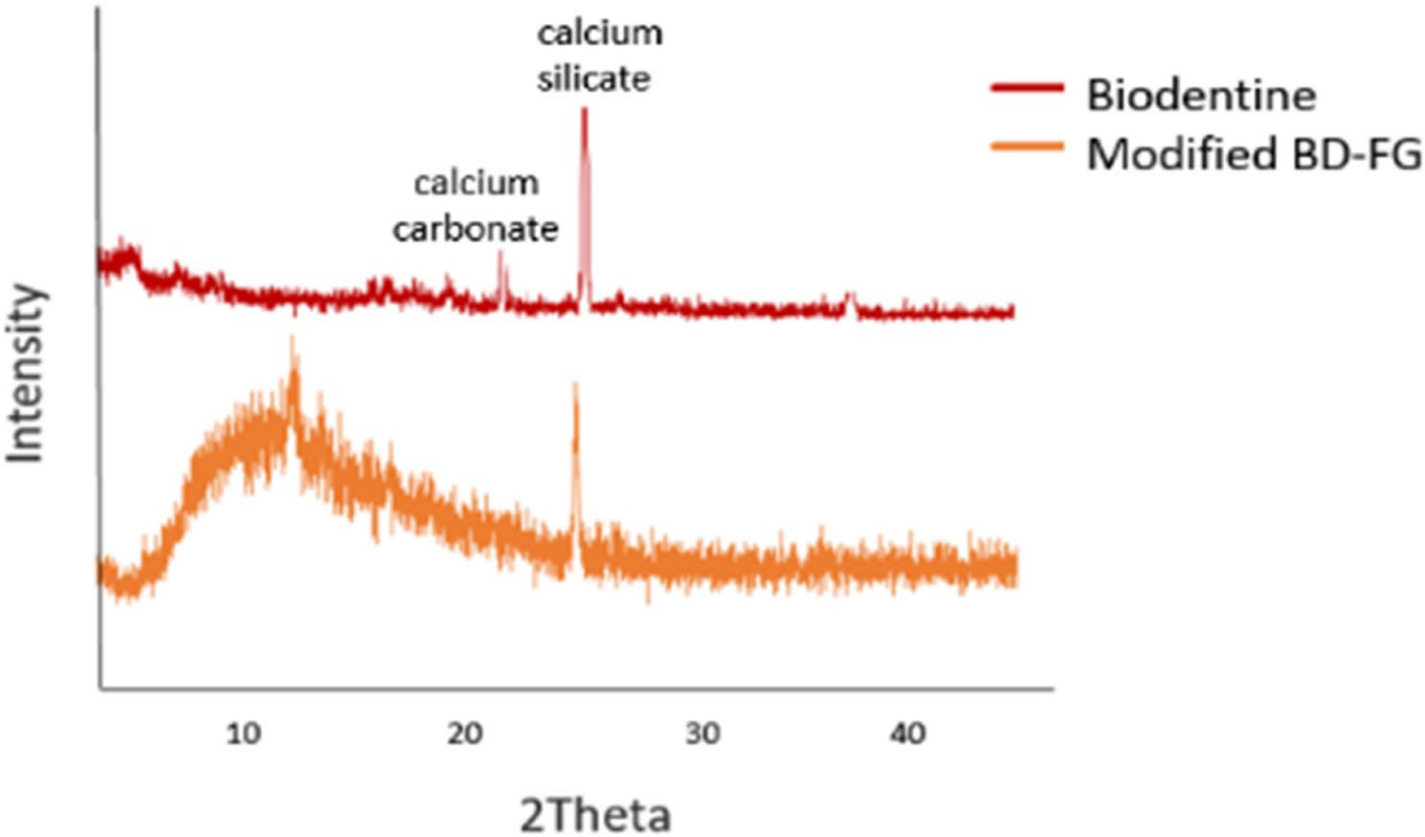
XRD patterns of Biodentine and modified BD-FG

XRD results (Fig. 4) exhibited strong peaks attributed to calcium carbonates And calcium silicates of Biodentine at approximately 2θ=23.5 ° And 26.3°, respectively. However, after modification of Biodentine with FG, a broad peak appeared at the region of 2θ= 10°-13° approximately, which is attributed to the strong peak of the 001-plane associated with graphene nanosheets.

**Fig. 5:**
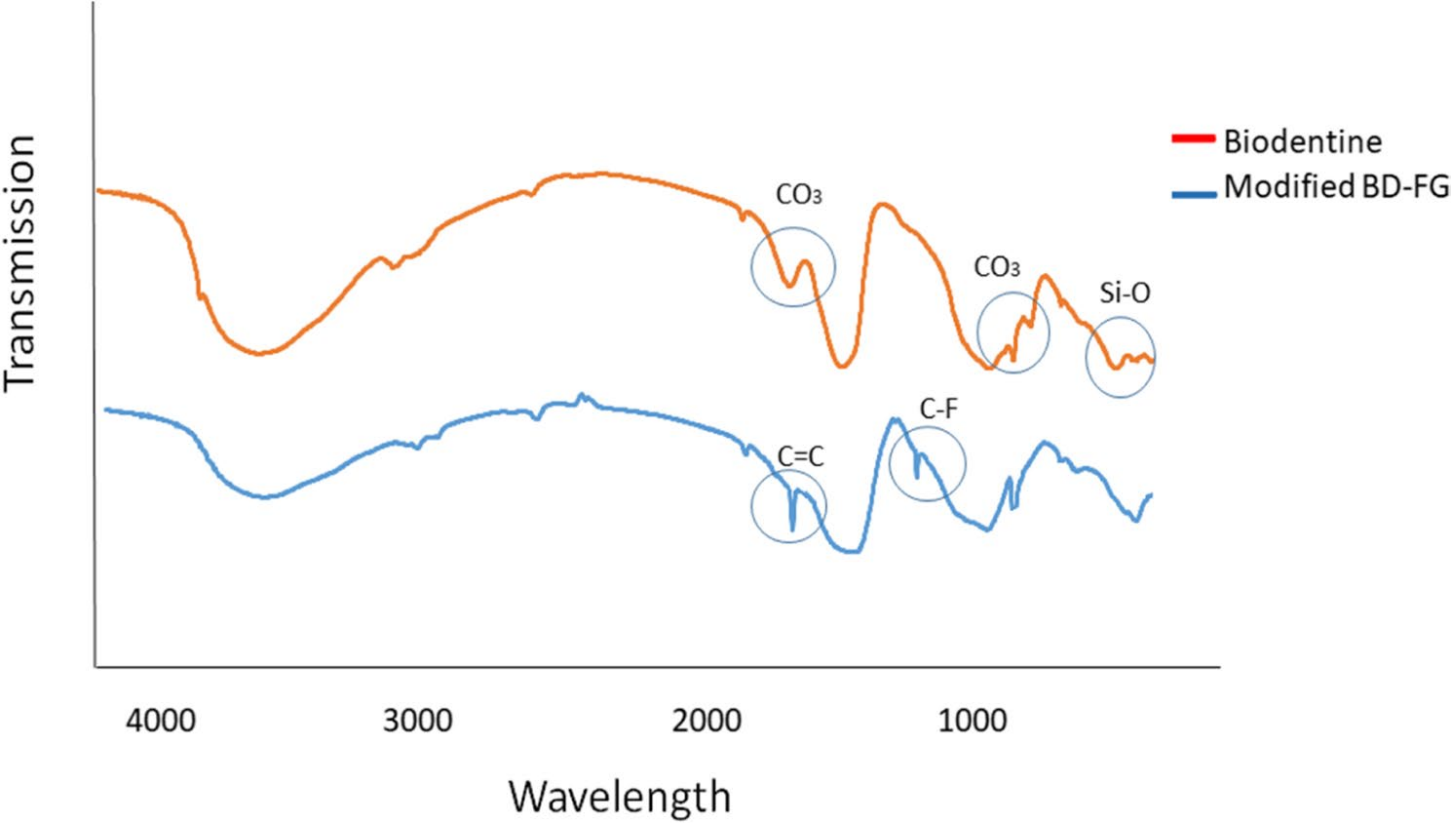
FTIR spectra of Biodentine and modified BD-FG

FTIR analysis of Biodentine and modified Biodentine (BD-FG) (Fig. 5) revealed bands at approximately 1780, 900, And 800 cm^-1^ attributed to CO_3_ bonds associated with calcium carbonates, in addition to bands at approximately 500 cm-1attributed to silica vibrations. FTIR of modified BD-FG displayed new peaks of C-F and C=C bonds associated with graphene at about 1200 cm^-1^ And 1700 cm^-1,^ respectively.

**Fig. 6:**
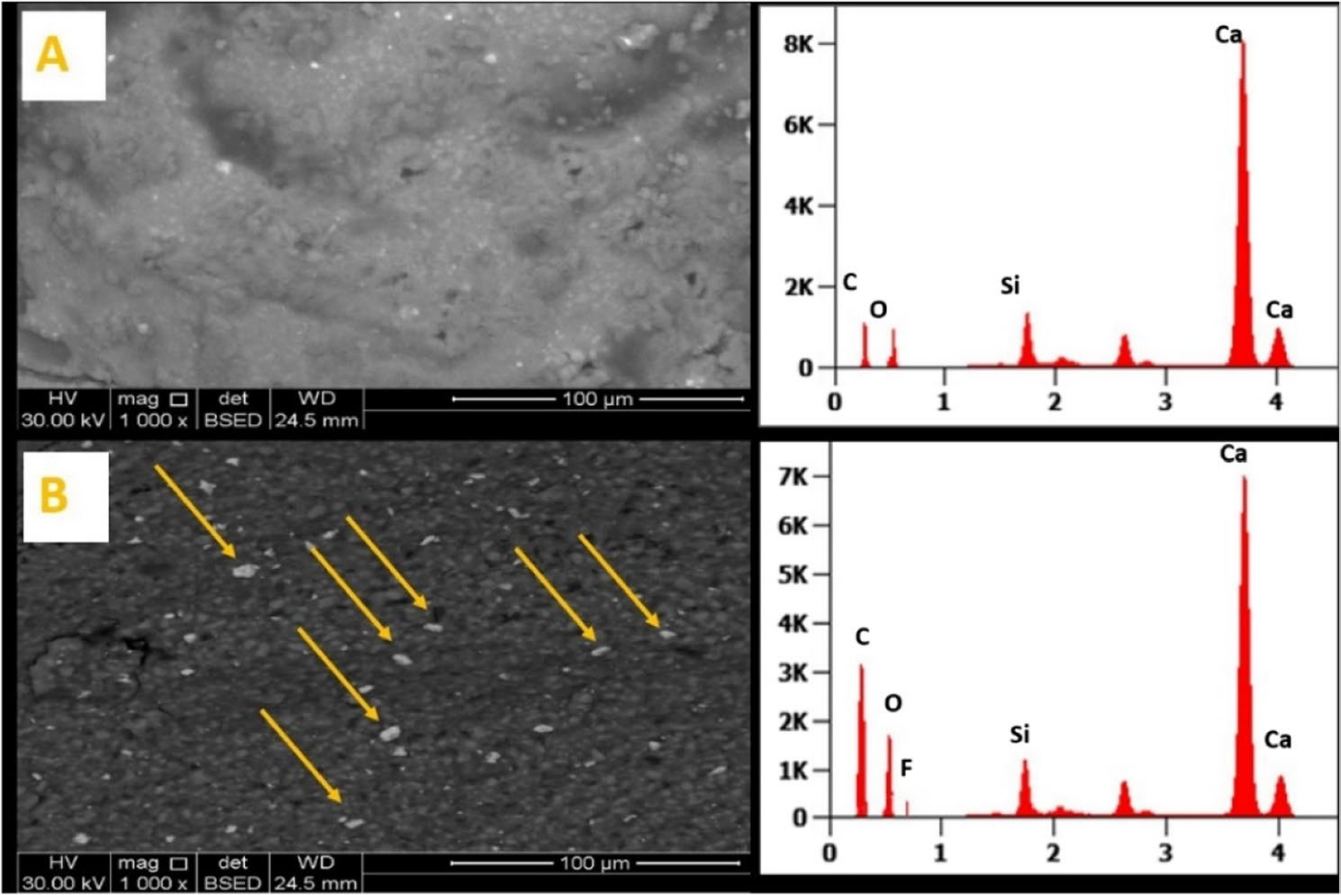
SEM images and EDX analysis of (**A**) unmodified Biodentine and (**B**) modified BD-FG, arrows show FG particles

**Fig. 7:**
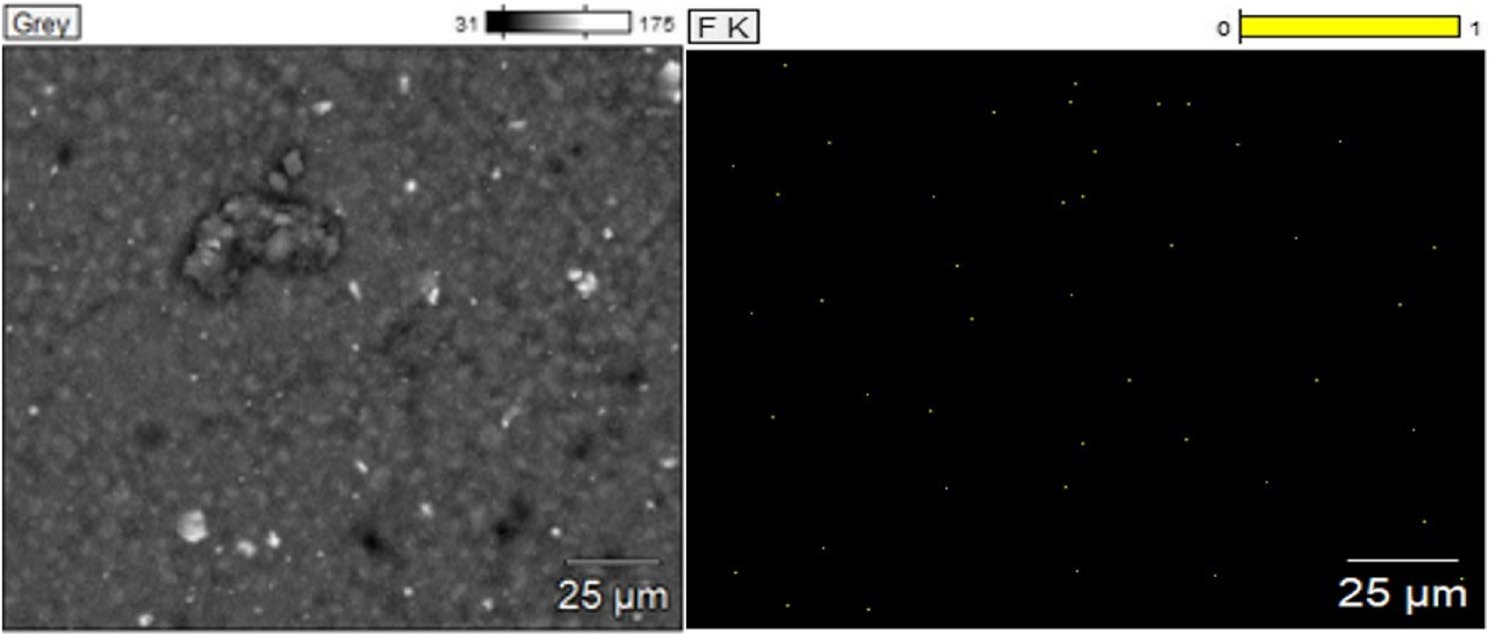
Elemental mapping of fluoride in the modified BD-FG group

SEM imaging (magnification 1000X) of unmodified BD (Fig. 6A) revealed homogenous and smooth surface morphology. However, the BD-FG group revealed a rough and porous surface (Fig. 6B). EDX analysis results (Fig. 6 and Table 1) showed the elemental composition of the BD and BD-FG groups. The results showed that oxygen and carbon percentages were increased in the BD-FG group, while calcium and silicon percentages were decreased, with the appearance of the fluoride (F) element. F mapping results revealed uniform distribution of FG in Biodentine (Fig. 7).

**Table 1:** Elemental atomic% of unmodified BD and BD-FG obtained by EDX analysis

Elements	Atomic %	
	Unmodified BD group	Modified BD-FG group
Oxygen	36.8	33.8
Calcium	50.7	45.6
Silicon	6.9	5.6
Carbon	5.6	14.3
Fluoride	0	0.7
Total	100%	100%

### Ph behavior and ion release results

Two-way ANOVA results showed that the type of BD had no statistically significant effect on pH at p=0.39, however, it revealed a statistically significant effect on Ca ion release at p<0.001. The immersion time showed a statistically significant effect on both pH and Ca ion release (p = 0.007 and p<0.001, respectively). The interaction between the two variables had a statistically significant effect on pH and Ca ion release (p=0.007, p<0.001, respectively).

**Fig. 8:**
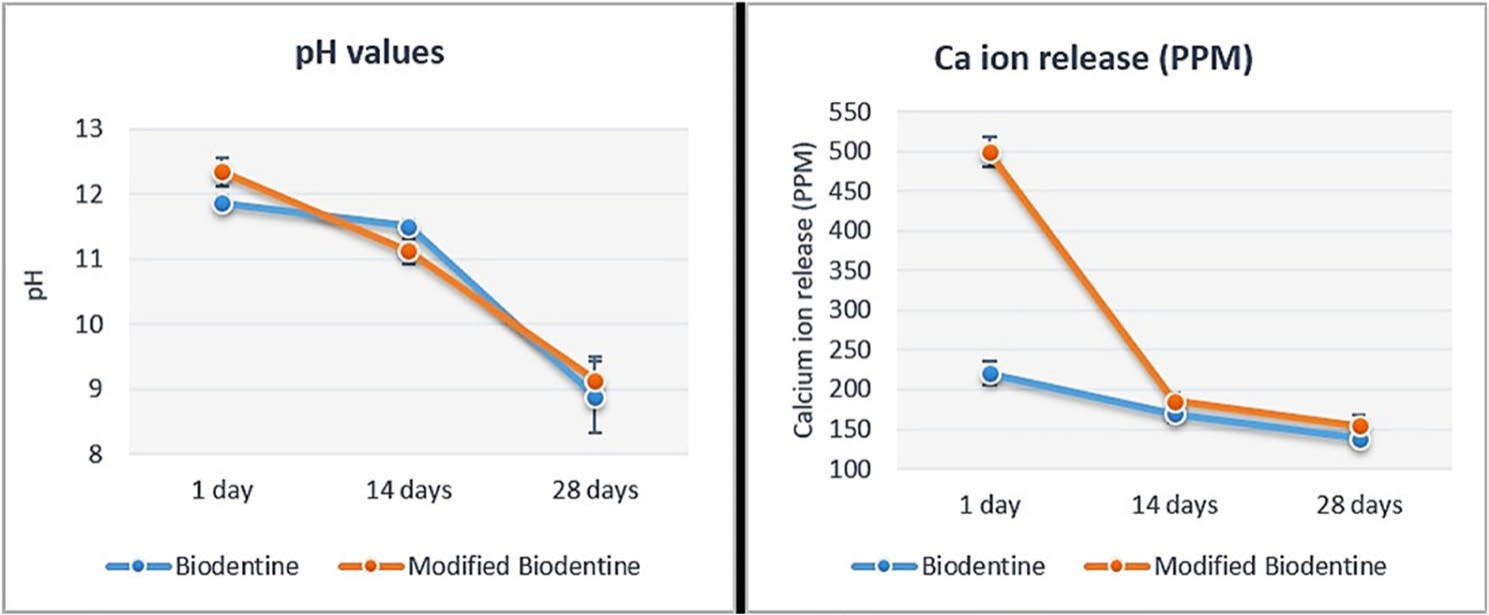
Line chart showing pH and calcium ion release mean values (PPM)

**Fig. 9:**
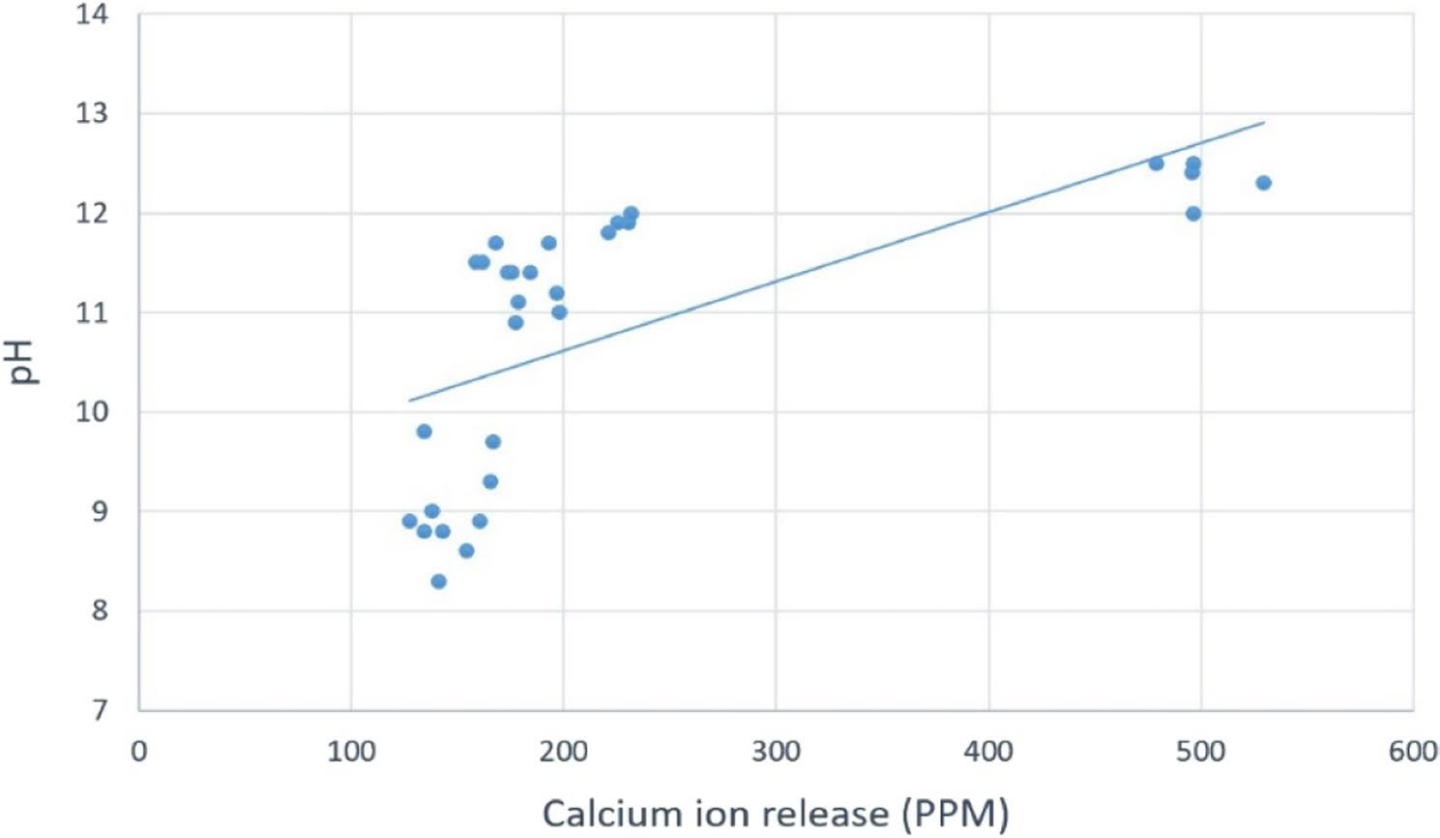
Scatter plot showing Ca ion release and pH correlation

Statistical Analysis results revealed that the modified BD-FG group exhibited a significantly higher pH And Ca ion release at day 1 (p=0.021 and p<0.01, respectively). However, no statistically significant difference was found between the groups at days 3 And 7 (Fig. 8). It was also noticed that pH and calcium ion release of both groups exhibited a descending pattern with time. In addition, there was a prominent positive correlation between Ca ion release and pH that was statistically significant (p<0.001) (Fig. 9).

**Fig. 10:**
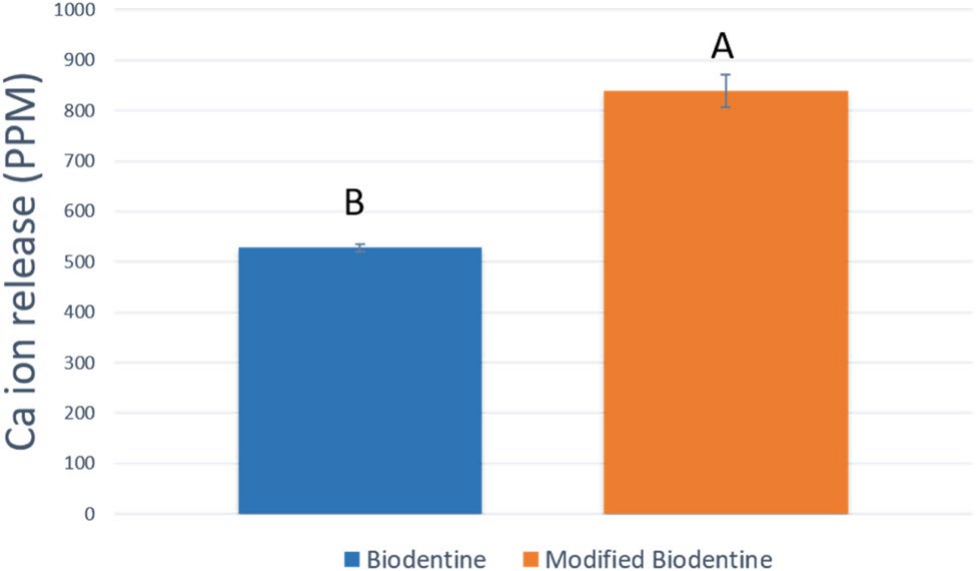
Bar chart showing the cumulative calcium ion release mean (PPM), standard deviation and statistical significant difference

Regarding cumulative calcium ion release (Fig. 10),statistical analysis revealed that the modified BD-FG group recorded a significantly higher mean value compared to the other group (P<0.001).

**Fig. 11:**
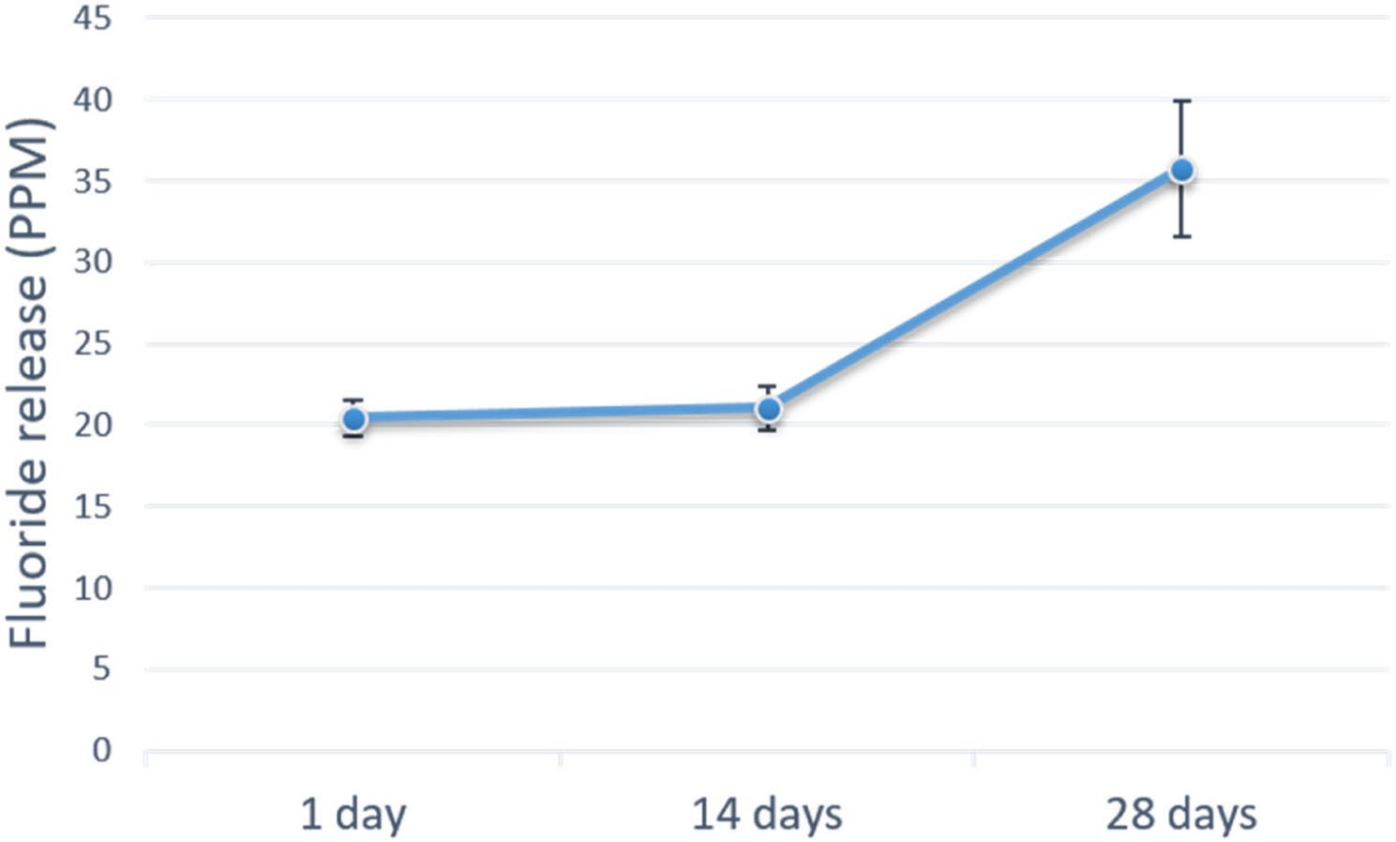
Line chart showing fluoride ion release mean (PPM) and standard deviation

The results of fluoride ion release from the modified BD-FG group (Fig. 11) revealed a significant difference in the amount of fluoride released over time (p < 0.001). The results of the pairwise comparisons revealed a significant increase in fluoride after 28 days compared to measurements taken at day 1 And day 14. The cumulative fluoride release after 28 days was (77.26 ± 4.88 PPM).

### In vitro apatite formation ability results

**Fig. 12:**
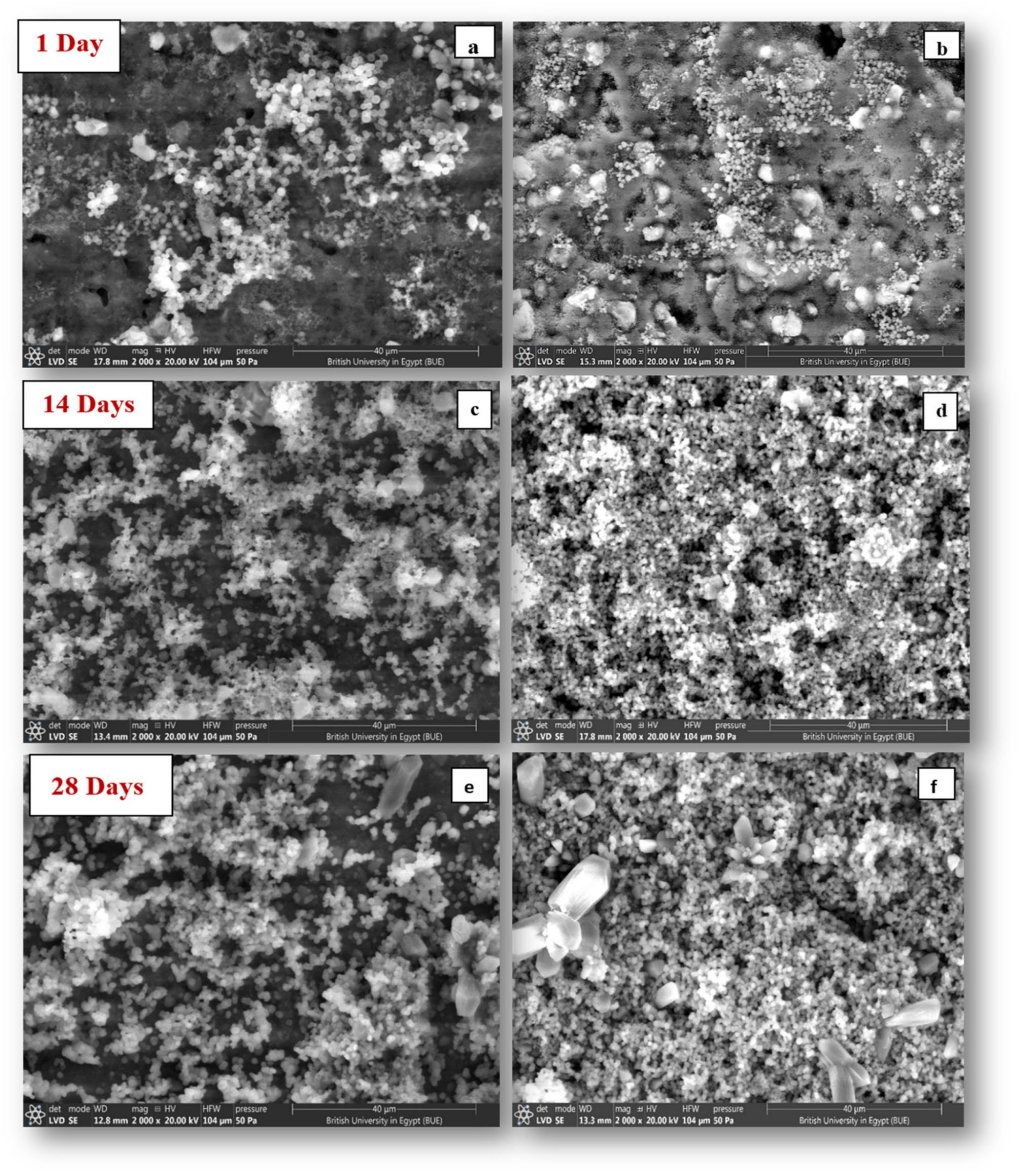
ESEM micrographs at magnification 2000X showing surface deposits over unmodified BD (**a**,** b**, **c**) and modified BD-FG (**d**, **e**,** f**) at different time intervals

The results of ESEM (Fig. 12) showed progressive formation of superficial spherules on the surfaces of both groups that increased with the increase in immersion time from day 1 to day 28. Precipitates formed on the BD-FG group's surface appeared to be much denser than the unmodified BD group for each time interval.

**Fig. 13:**
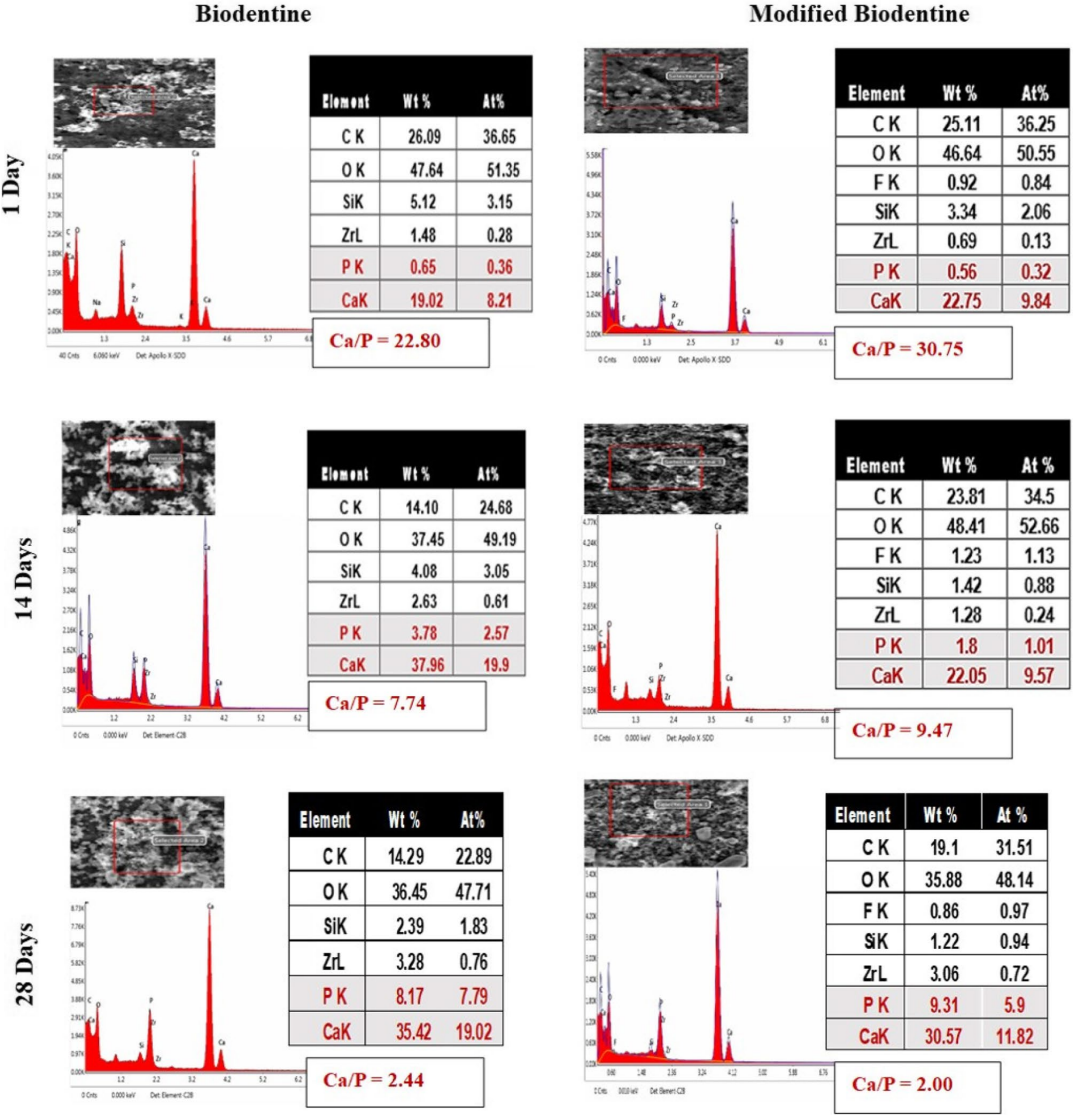
EDX spectral analysis of unmodified BD and modified BD-FG at different time intervals

EDX analysis (Fig. 13) showed peaks of calcium, silicon, carbon, and oxygen, denoting the different reaction phases present in the set cement. In addition, the presence of peaks of calcium and phosphorus denoted the formation of the apatite layer precursor. Results also exhibited a higher Ca/P ratio in the modified BD-FG group compared to the unmodified group. However, both groups displayed a decrease in the calcium/phosphorus ratio over time.

### Cell viability results

**Fig. 14:**
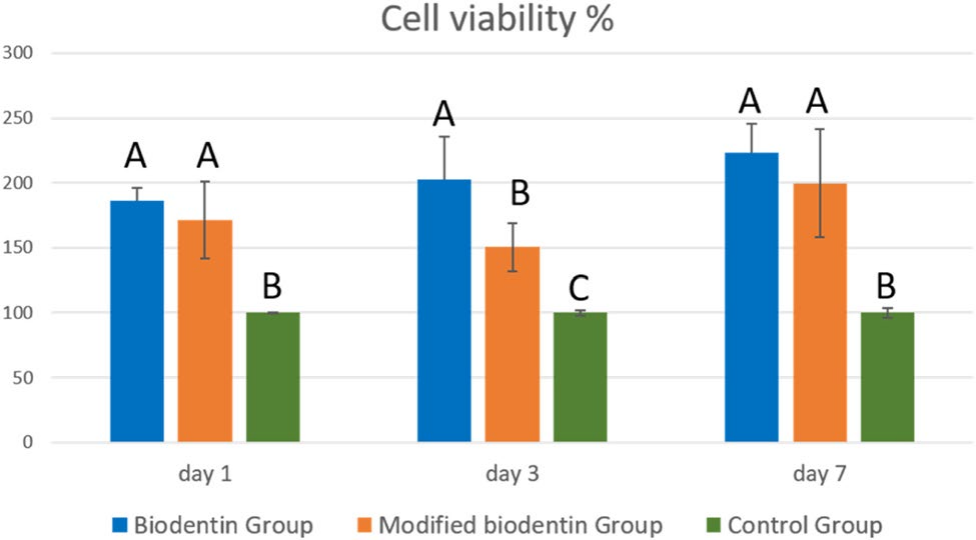
Bar chart showing cell viability % results, standard deviation and statistical significant difference between different groups at each time interval

Statistical results of MTT assay revealed that both the modified and unmodified Biodentine groups showed significantly higher cell viability % compared to the control group at all tested time intervals (p<0.001) (Fig. 14). The statistical results also revealed no statistically significant difference between modified And unmodified Biodentine groups at day 1 And day 7. However, at day 3, the unmodified BD group showed significantly higher cell viability % compared to the modified BD-FG group (p<0.001).

All tested materials demonstrated non-cytotoxic behavior at all tested time intervals, with cell viability exceeding 80% compared to the control group. On day 1, Biodentine And modified Biodentine groups showed viabilities of approximately 186% And 172% respectively. This trend continued at day 3 And day 7, with Biodentine maintaining the highest viability (202% And 228%), followed by modified Biodentine (150% And 185%). The control group showed a gradual increase in viability over time, reaching 124% on day 7. These results confirm the biocompatibility of both materials and suggest a time-dependent increase in cellular metabolic activity.

### Alkaline phosphatase assay result

**Fig. 15:**
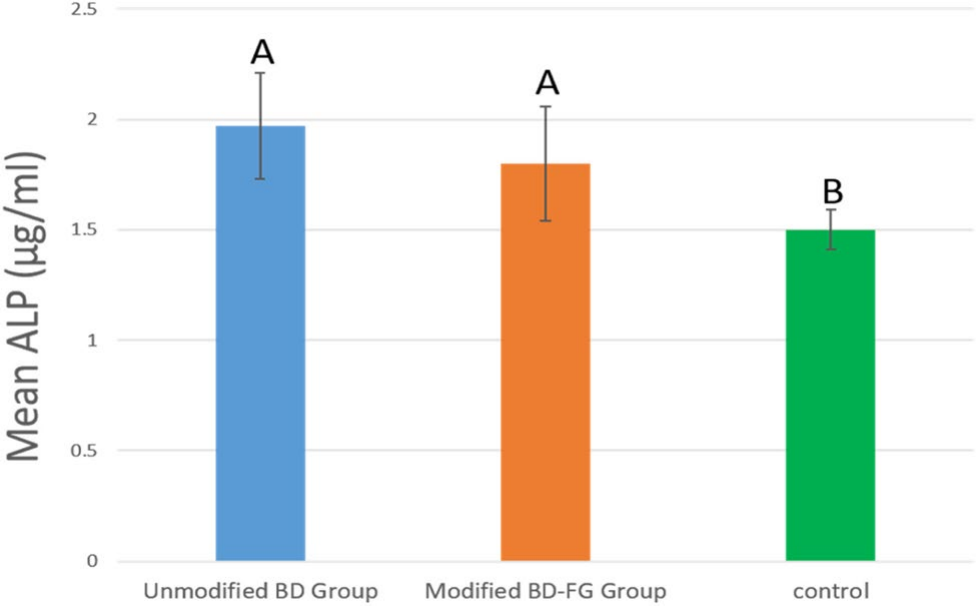
Bar chart showing ALP mean values (μg/mL), standard deviation And statistical significant difference between different groups at day 7

Statistical analysis revealed that both modified and unmodified Biodentine groups showed significantly higher ALP activity compared to the control group (p<0.001) (Fig. 15). However, the results showed no statistically significant difference between modified and unmodified Biodentine groups (P=0.087).

**Fig. 16:**
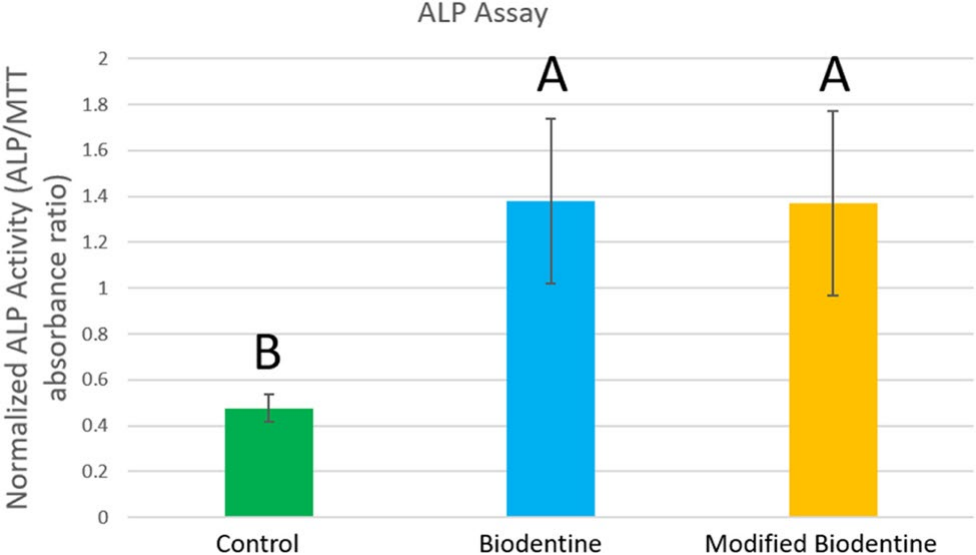
16 Bar chart showing normalized ALP activity to cell viability, standard deviation and statistical significant difference between different groups. Normalized ALP values were expressed as ALP absorbance per unit of viable cell absorbance (ALP/MTT)

Both Biodentine and modified Biodentine groups demonstrated significantly higher normalized ALP activity compared to the control (p<0.01), indicating enhanced osteogenic potential (Fig. 16 and table 2). No statistical significant difference was observed between Biodentine and modified Biodentine groups, suggesting that the modification did not further augment ALP expression under the conditions tested.

**Table 2:** Normalized ALP activity relative to cell viability; mean, standard deviation and statistical significance between different groups

Group	Normalized ALP/MTT	SD ±	Significance vs. Control	F-value	*P*- value
Control	0.477	0.05	—	9.895004	0.012592
Biodentine	1.38	0.2	*p* < 0.01		
Modified Biodentine	1.37	0.18	*p* < 0.01		

### Compressive strength results

Statistical analysis revealed that the unmodified BD group (102.82 ± 7.78) exhibited significantly lower compressive strength compared to the modified BD-FG group (123.74 ± 7.07) (P <0.001)

**Fig. 17:**
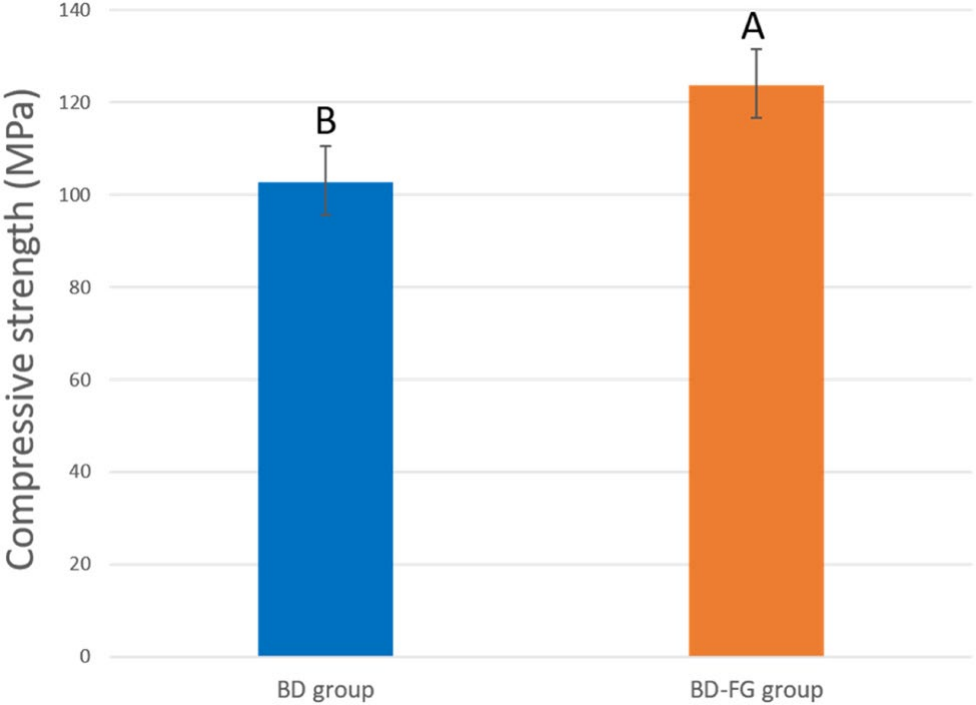
Bar chart showing compressive strength mean (MPa), standard deviation and statistical significant difference between different groups

## Discussion

The present study evaluated the effect of adding fluorinated graphene for the modification of Biodentine, which has been frequently acknowledged in literature for its superior physical properties, good handling characteristics, and wide range of clinical applications. In the current study, fluorinated graphene was prepared from graphite using modified Hummers’ methodology, as it is reported to be the most commonly used, reliable method [24]. The prepared FG powder was characterized using XRD, FTIR, and TEM. XRD spectrum showed the planes (001), (002), and (100) of graphene sheets (Fig. 1). FTIR spectrum further confirmed the XRD findings, as it also displayed the strong peak of C-F group (Fig. 1), which is usually used to identify F in FG. The stretching vibration of C-F2 at the nanosheet margin was also detected. Moreover, the existence of a graphene basal plane of C = C was also confirmed. The stretching vibration peaks of O-H groups correspond to oxygen functional groups [26, 27]. The lamellar nano-sheet morphology of FG was further confirmed by TEM analysis (Fig. 3).

Based on a pilot study, the addition of FG into Biodentine enhanced the mechanical properties of Biodentine up to 2 wt% of FG, suggesting that up to this concentration limit, the FG didn’t compromise Biodentine properties. Accordingly, a concentration of 2 wt% FG powder was selected to modify Biodentine in order to prepare FG-modified Biodentine (BD-FG group).

Biodentine before And after modification with 2 wt% FG was characterized by XRD, FTIR, and SEM-EDX. Both XRD and FTIR results showed peaks associated with fluorinated graphene nanosheets confirming the preparation of modified BD-FG (Figs. 2 And 5 respectively). SEM image of BD-FG group showed rougher and more porous surface due to the incorporation of nanoparticles (Fig. 3). This agreed with a study by Koutroulis et al. [28]. EDX analysis of both groups showed high percentages of Ca, O, Si, and C, related to the composition of Biodentine, which is mainly tricalcium and calcium carbonate [3, 5]. EDX results of the BD-FG group revealed increased C concentration, as well as displayed F concentration (Table 1). This is related to the incorporated FG, which showed homogenous dispersion within Biodentine (Fig. 4).

Calcium and hydroxide ions release is considered a key success factor of the material’s bioactivity, as they stimulate the differentiation of hard tissue-forming cells, hence improving mineralization. So, it was of prime importance to evaluate calcium ion release values and to assess the amount of eluted hydroxide ions by measuring the pH [29]. Hanks’ Balanced Salt Solution (HBSS) was used in this study due to its good ability to induce in vitro apatite formation, as well as its ability to simulate the ionic concentration of the human blood plasma [30]. The results of the current study revealed that both groups exhibited alkaline pH throughout all the tested time intervals, which indicates a favorable condition to maintain high osteogenic potential. The results showed that the modified BD-FG group exhibited significantly higher pH And Ca ion release values at day 1 (Fig. 8). It was noticed from SEM images that BD-FG showed a more porous structure (Fig. 3), which could lead to high fluid uptake, and hence, increase its initial solubility, and accelerate its hydration kinetics. Accordingly, more hydroxyl ions were released, which increased alkalinity, and more Ca ions were released, which favored rapid mineralization. This could also explain its significantly higher cumulative Ca ions release (Fig. 6).

The results of the current study also showed fluoride ion release from the modified BD-FG group, which exhibited An increase over time, with a significantly higher value at 28 days (Fig. 7). Fluoride ion release could favor enamel remineralization, limit bacterial enzymes involved in acid production, ultimately strengthening teeth and developing an anti-cariogenic effect [31].

Imaging the material’s surface by ESEM in conjunction with EDX was used to analyze the elemental composition of the surfaces of the tested materials after storage in HBSS. In this study, ESEM analysis was performed instead of the conventional SEM to avoid any surface preparation or coating that could cause any change in the surface chemistry. ESEM results (Fig. 8) revealed the presence of superficial precipitates of various sizes on the surfaces of both groups that increased in size over time. EDX results revealed that Ca and P were the main constituents of the formed precipitates, suggesting the formation of an apatite layer (Fig. 13). In fact, the bioactivity of Biodentine occurs through the hydration of the tricalcium silicate and the formation of calcium hydroxide, which in the presence of phosphate ions precipitate into a hydroxyapatite mineral [32]. It was observed that the modified BD-FG group displayed thicker and densely dispersed mineralized precipitates compared to the other group at all tested time intervals (Fig. 8). This could be related to its accelerated hydration kinetics and its higher ion release. According to Xu et al., the incorporation of graphene in calcium silicate cement provides more nucleation sites for calcium silicate hydrate, because it is more negatively charged, so it attracts more positively charged calcium ions, thus accelerating the deposition of minerals [33].

EDX results revealed that both groups exhibited an early higher Ca/P ratio, which indicates an early nucleation of Ca-rich apatite precursors. However, the Ca/P ratio of both groups gradually decreased over time, owing to the increase in phosphate precipitation, indicating the maturity of the apatite layer. This agreed with the previous study [34].

Cell viability results of this study demonstrated that both modified and unmodified Biodentine groups exhibited significantly higher cell viability compared to the control group at all tested time intervals (*p* < 0.001) (Fig. 9), indicating the ability of both groups to promote pulp cell proliferation. The results showed no statistically significant difference between the modified And unmodified Biodentine groups at days 1 And 7. However, the modified BD-FG group exhibited significantly lower cell viability at day 3 compared to the unmodified BD group (*p* < 0.001). A previous study speculated an increase in apoptotic and necrotic cells with increasing extracellular mineralization induced by calcium ion release in cultured human dental pulp stem cells [35]. Other study also reported the cytotoxic effect of high calcium content [36]. Accordingly, it could be suggested that the higher ion release of the BD-FG group, could contribute to decreasing the cell viability at day 3. Nevertheless, it was noticed that the cell viability of the BD-FG group has been significantly increased at day 7, which could be related to the noticed decline pattern of Ca ion release over time. This could suggest the ability of the cells to recover and resume proliferation. According to Geng et al., as the incubation period of cells with FG increases, the intracellular oxidative stress levels increase, which further exerts an adverse influence on cellular functions [37]. This also agreed with other study [38]. However, some previous studies reported the ability of FG to enhance mesenchymal stem cells’ adhesion, improve their proliferation, and support their differentiation [39–41]. The discrepancies between cell viability findings in different studies could be due to differences in graphene concentration, degree of fluorination, and details on shape, size, and dispersion. In addition to variations in the nature of the modified biomaterial.

ALP results came following MTT assay results. As, no statistically significant difference was found between the two groups in the ALP activity at day 7 (Fig. 10). In fact, ALP expression is one of the most commonly used markers to assess odontogenic differentiation of dental pulp stem cells, and it is related to the process of mineral deposition; hence, it plays a significant role in pulp repair mechanisms [29]. Accordingly, it could be emphasized that FG didn’t compromise odontogenic differentiation and extracellular mineral deposition when added into BD.

Compressive strength is an important property for hydraulic cements in vital pulp therapies, so that they can withstand masticatory forces. Compressive strength results showed that BD-FG exhibited a significantly higher compressive strength compared to the BD group (Fig. 17). Many previous studies revealed that adding carbon-based materials to calcium silicate cements could contribute to improved mechanical properties [14, 42, 43]. This mechanical enhancement could be related to the intrinsic high modulus of elasticity and strength of FG [19]. Moreover, FG nanosheets have lower surface energy, which facilitates their good dispersion within BD (as shown in Fig. 4) [17], allowing them to act as crack deflectors and preventing crack propagation [19]. Another explanation was stated by Xu et al., who found out that graphene enhanced mineral deposition not only on the cement surface but also within the pores of the cement, which resulted in decreasing porosity and increasing strength [33].

This study introduced a novel modification of Biodentine using 2 wt% fluorinated graphene (FG), offering a promising strategy to enhance some properties of Biodentine. The employment of different characterization techniques -XRD, FTIR, TEM, and SEM/EDX- showed in-depth analysis of structural, morphological, and elemental properties of the new tested material. Moreover, the study incorporates physicochemical, mechanical, and biological evaluations, providing a holistic understanding of the modified material’s performance. The use of stem cells derived from dental pulp further enhances the clinical relevance of the biological assessments. However, this study has several limitations that should be acknowledged. Properties including setting time, solubility, water sorption, antibacterial, sealing ability, and bond strength, were not evaluated in this study and are recommended to be considered in further studies. Besides, other commercially available protein assays like bicinchoninic acid (BCA) assay or Bradford assay are also recommended to be used in future studies, providing an additional option for ALP normalization. Moreover, the effect of different concentrations of FG also needs to be examined. Additionally, in vivo examinations of FG-modified biomaterials should also be taken into consideration in future research.

## Conclusion

Within the limitations of this study, it could be concluded that FG is a promising nanofiller that could be added into Biodentine to increase Ca ion release, promote F ion release, in addition to improving mechanical properties without compromising cell viability and differentiation potential.

## Supplementary Information

Supplementary Material 1.

## Data Availability

No datasets were generated or analysed during the current study.

## Abbreviations

BD: Biodentine
FG: fluorinated graphene
MTA: Mineral trioxide aggregate
XRD: X-ray diffraction
FTIR: Fourier Transform Infrared Spectra
SEM: Scanning electron microscope
TEM: Transmission electron microscope
EDX: Energy dispersive x-ray spectroscopy
ESEM: Environmental scanning electron microscope
ALP: Alkaline phosphatase

## References

[1] Singla MG, Wahi P. Comparative evaluation of shear bond strength of biodentine, endocem mineral trioxide aggregate, and theracal LC to resin composite using a universal adhesive: an in vitro study. Endodontology. 2020;32:14. 10.4103/endo.endo_7_19.

[2] Nie E, Yu J, Jiang R, Liu X, Li X, Islam R, et al. Effectiveness of direct pulp capping bioactive materials in dentin regeneration: a systematic review. Materials. 2021;14:6811. 10.3390/ma14226811. 34832214 10.3390/ma14226811 PMC8621741

[3] Kaur M, Singh H, Dhillon JS, Batra M, Saini M. MTA versus biodentine: review of literature with a comparative analysis. J Clin Diagn Res. 2017;11(8):ZG01. 10.7860/JCDR/2017/25840.10374. 28969295 10.7860/JCDR/2017/25840.10374 PMC5620936

[4] Mustafa RM, Al-Nasrawi SJ, Aljdaimi AI. The effect of biodentine maturation time on resin bond strength when aged in artificial saliva. Int J Dent. 2020. 10.1155/2020/8831813. 33144858 10.1155/2020/8831813 PMC7599420

[5] Batul R, Makandar SD, Nawi MABA, Basheer SN, Albar NH, Assiry AA, et al. Comparative evaluation of microhardness, water sorption, and solubility of Biodentin and nano-zirconia-modified Biodentin and FTIR analysis. Appl Sci. 2023;13:1758. 10.3390/app13031758.

[6] Guneser MB, Ozturk TY, Sahin AND, Uysal BA, Eldeniz AU. Effect of nanosized bioactive glass addition on some physical properties of Biodentine. J Appl Biomater Funct Mater. 2023;21:22808000231184059. 10.1177/22808000231184059. 37680087 10.1177/22808000231184059

[7] Fagr E, Ola E, Dina W. In vitro evaluation of shear bond strength of polymethyl methacrylate/montmorillonite modified Biodentine with dental resin composite. Eur Oral Res. 2025;1(1):19–26. 10.26650/eor.20241339433. 10.26650/eor.20241339433 PMC12126164 40453405

[8] Fathy SM, Abd El-Aziz AM, Maaly TM, Elhendawi H, Elkhooly TA. Effect of biotitania and titania addition on bioactivity and antibacterial properties of calcium silicate cement. Iran Endod J. 2020;15(3):173. 10.22037/iej.v15i3.28490. 36703805 10.22037/iej.v15i3.28490 PMC9709847

[9] Sharma N, Yadav D, Hassan MI, Srivastava CM, Majumder S. A review on exploring the impact of graphene oxide-based nanomaterials on structures and bioactivity of proteins. J Mol Liq. 2024;124980. 10.1016/j.molliq.2024.124980.

[10] Dubey N, Bentini R, Islam I, Cao T, Castro Neto AH, Rosa V. Graphene: a versatile carbon-based material for bone tissue engineering. Stem Cells Int. 2015;2015(804213). 10.1155/2015/804213. 10.1155/2015/804213 PMC4466492 26124843

[11] Hosseini FS, Laurencin CT. Advanced graphene ceramics and their future in bone regenerative engineering. Int J Appl Ceram Technol. 2022;19:893–905. 10.1111/ijac.13999.

[12] AlFawaz YF, Almutairi B, Kattan HF, Zafar MS, Farooq I, Naseem M, et al. Dentin bond integrity of hydroxyapatite containing resin adhesive enhanced with graphene oxide nano-particles—an SEM, EDX, micro-Raman, and microtensile bond strength study. Polymers. 2020;12:2978. 10.3390/polym12122978. 33327410 10.3390/polym12122978 PMC7764838

[13] Mei L, Wei H, Wenjing C, Xiaokun H. Graphene oxide-silica composite fillers into the experimental dental adhesives for potential therapy. Med Res. 2017;1:42. 10.21127/yaoyimr20170012.

[14] Somaie RA, El-Banna A, El-Korashy DI. Effect of incorporation of nano-graphene oxide on physicochemical, mechanical, and biological properties of tricalcium silicate cement. J Mech Behav Biomed Mater. 2023;146:106078. 10.1016/j.jmbbm.2023.106078. 37597312 10.1016/j.jmbbm.2023.106078

[15] Mazánek V, Jankovský O, Luxa J, Sedmidubský D, Janoušek Z, Šembera F, Mikulics M, Sofer Z. Tuning of fluorine content in graphene: towards large-scale production of stoichiometric fluorographene. Nanoscale. 2015;7:13646–55. 10.1039/C5NR03243A. 26214601 10.1039/c5nr03243a

[16] Baldissara P, Silvestri D, Pieri GM, Mazzitelli C, Arena A, Maravic T, et al. Effect of fluorographene addition on mechanical and adhesive properties of a new core build-up composite. Polymers. 2022;14:5301. 10.3390/polym14235301. 36501696 10.3390/polym14235301 PMC9737195

[17] Chen X, Fan K, Liu Y, Li Y, Liu X, Feng W, Wang X. Recent advances in fluorinated graphene from synthesis to applications: critical review on functional chemistry and structure engineering. Adv Mater. 2022;34:2101665. 10.1002/adma.202101665. 10.1002/adma.202101665 34658081

[18] Aboelwafa MR, Shaheen SD. Microhardness, surface roughness, and wear resistance enhancement of reinforced conventional glass ionomer cement using fluorinated graphene oxide nanosheets. Eur J Dent. 2024;18:1116–23. 10.1055/s-0044-1785188. 38759994 10.1055/s-0044-1785188 PMC11479740

[19] Sun L, Yan Z, Duan Y, Zhang J, Liu B. Improvement of the mechanical, tribological, and antibacterial properties of glass ionomer cements by fluorinated graphene. Dent Mater J. 2018;34:e115–27. 10.1016/j.dental.2018.02.006. 10.1016/j.dental.2018.02.006 29567317

[20] Liu R, Wang E, Guo Y, Zhou Q, Zheng Y, Zhai J, et al. Enhanced antibacterial properties and promoted cell proliferation in glass ionomer cement by modified with fluorinated graphene-doped. J Appl Biomater Funct Mater. 2021;19:22808000211037487. 10.1177/22808000211037487. 34428976 10.1177/22808000211037487

[21] Arafa SK, Sherief DI, Nassif MS. Effect of aging on mechanical and antibacterial properties of fluorinated graphene reinforced glass ionomer: in vitro study. J Mech Behav Biomed Mater. 2023;142:105803. 10.1016/j.jmbbm.2023.105803. 37031564 10.1016/j.jmbbm.2023.105803

[22] Maryoosh RM, Al-Shamma AM. Shear bond strength of fluorinated graphene nanoparticles modified dental adhesives. Ann Trop Med Public Health. 2020;23:1–5. 10.36295/ASRO.2020.231373.

[23] Maryoosh RM, Al-Shamma A. Development and assessment of fluorinated grapheme nanoparticles modified dental adhesives. Ann Trop Med Public Health. 2020;20:41588. 10.37506/mlu.v20i4.2058.

[24] Marcano DC, Kosynkin DV, Berlin JM, Sinitskii A, Sun Z, Slesarev A, Alemany LB, Lu W, Tour JM. Improved synthesis of graphene oxide. ACS Nano. 2010;4:4806–14. 10.1021/nn1006368. 20731455 10.1021/nn1006368

[25] Aguilar-Bolados H, Contreras-Cid A, Yazdani-Pedram M, Acosta-Villavicencio G, Flores M, Fuentealba P, et al. Synthesis of fluorinated graphene oxide by using an easy one-pot deoxyfluorination reaction. J Colloid Interface Sci. 2018;524:219–26. 10.1016/j.jcis.2018.04.030. 29655140 10.1016/j.jcis.2018.04.030

[26] Li H, Hou G, Tian Q, Bi S, Liu Z, Lin Y, Tang J, Su X. Preparation of fluorinated graphene oxide with different oxygen content and its superhydrophobic property. Mater Res Express. 2019;6:045601. 10.1088/2053-1591/aaf8da.

[27] Valencia C, Valencia CH, Zuluaga F, Valencia ME, Mina JH, Grande-Tovar CD. Synthesis and application of scaffolds of chitosan-graphene oxide by the freeze-drying method for tissue regeneration. Molecules. 2018;23:2651. 10.3390/molecules23102651. 30332775 10.3390/molecules23102651 PMC6222393

[28] Koutroulis A, Valen H, Ørstavik D, Kapralos V, Camilleri J, Sunde PT. Surface characteristics and bacterial adhesion of endodontic cements. Clin Oral Investig. 2022;26:6995–7009. 10.1007/s00784-022-04655-y. 35931891 10.1007/s00784-022-04655-y PMC9708781

[29] Kang S. Mineralization-inducing potentials of calcium silicate-based pulp capping materials in human dental pulp cells. Yeungnam Univ J Med. 2020;37:217–25. 10.12701/yujm.2020.00248. 32438533 10.12701/yujm.2020.00248 PMC7384909

[30] Baino F, Yamaguchi S. The use of simulated body fluid (SBF) for assessing materials bioactivity in the context of tissue engineering: review and challenges. Biomimetics. 2020;5:57. 10.3390/biomimetics5040057. 33138246 10.3390/biomimetics5040057 PMC7709622

[31] Kaup M, Schäfer E, Dammaschke T. An in vitro study of different material properties of Biodentine compared to proroot MTA. Head Face Med. 2015;11:1–8. 10.1186/s13005-015-0074-9. 25934270 10.1186/s13005-015-0074-9 PMC4424823

[32] Paula A, Laranjo M, Marto CM, Abrantes AM, Casalta-Lopes J, Gonçalves AC, et al. Biodentine™ boosts, WhiteProRoot^®^ MTA increases, and Life^®^ suppresses odontoblast activity. Materials. 2019;12:1184. 10.3390/ma12071184. 30978943 10.3390/ma12071184 PMC6479701

[33] Xu C, Ma B, Peng J, Gao L, Xu Y, Huan Z, et al. Tricalcium silicate/graphene oxide bone cement with photothermal properties for tumor ablation. J Mater Chem B. 2019;7:2808–18. 10.1039/C9TB00246D. 32255083 10.1039/c9tb00246d

[34] Al-Sherbiny IM, Farid MH, Abu-Seida AM, Motawea IT, Bastawy HA. Chemico-physical and mechanical evaluation of three calcium silicate-based pulp capping materials. Saudi Dent J. 2021;33:207–14. 10.1016/j.sdentj.2020.02.001. 34025083 10.1016/j.sdentj.2020.02.001 PMC8119770

[35] An S, Gao Y, Huang Y, Jiang X, Ma K, Ling J. Short-term effects of calcium ions on the apoptosis and onset of mineralization of human dental pulp cells in vitro and in vivo. Int J Mol Med. 2015;36:215–21. 10.3892/ijmm.2015.2218. 25999211 10.3892/ijmm.2015.2218 PMC4494572

[36] Sabandal MMI, Schäfer E, Petsching S, Jung S, Kleinheinz J, Sielker S. Pleiotropic effects on proliferation and mineralization of primary human adipose tissue-derived stromal cells induced by simvastatin. Open Biol. 2022;12:210337. 10.1098/rsob.210337. 35673853 10.1098/rsob.210337 PMC9174717

[37] Geng H, Wang T, Cao H, Zhu H, Di Z, Liu X. Antibacterial ability, cytocompatibility and hemocompatibility of fluorinated graphene. Colloids Surf B Biointerfaces. 2019;173:681–8. 10.1016/j.colsurfb.2018.10.050. 30384264 10.1016/j.colsurfb.2018.10.050

[38] Sukumar T, Varghese J, Bhargavan S, Jayasree P, Suvekbala V, Alaganandam K, Ragupathy L. Cytotoxicity of formulated graphene and its natural rubber nanocomposite thin film in human vaginal epithelial cells: an influence of noncovalent interaction. ACS Biomater Sci Eng. 2020;6:2007–19. 10.1021/acsbiomaterials.9b01897. 32309635 10.1021/acsbiomaterials.9b01897 PMC7157971

[39] Wang Y, Lee WC, Manga KK, Ang PK, Lu J, Liu YP, Lim CT, Loh KP. Fluorinated graphene for promoting neuro-induction of stem cells. Adv Mater. 2012;24:4285. 10.1002/adma.201200846. 22689093 10.1002/adma.201200846

[40] Ahmad Y, Batisse N, Chen X, Dubois M. Preparation and applications of fluorinated graphenes. C. 2021;7:20. 10.3390/c7010020.

[41] Lammel T, Boisseaux P, Fernández-Cruz M-L, Navas JM. Internalization and cytotoxicity of graphene oxide and carboxyl graphene nanoplatelets in the human hepatocellular carcinoma cell line hep G2. Part Fibre Toxicol. 2013;10:1–21. 10.1186/1743-8977-10-27. 23849434 10.1186/1743-8977-10-27 PMC3734190

[42] Kucukyildiz EN, Dayi B, Altin S, Yigit O. In vitro comparison of physical, chemical, and mechanical properties of graphene nanoplatelet added Angelus mineral trioxide aggregate to pure Angelus mineral trioxide aggregate and calcium hydroxide. Microsc Res Tech. 2021;84:929–42. 10.1002/jemt.23654. 33410148 10.1002/jemt.23654

[43] Mehrali M, Moghaddam E, Seyed Shirazi SF, Baradaran S, Mehrali M, Latibari ST, Metselaar HSC, Kadri NA, Zandi K, Osman NAA. Mechanical and in vitro biological performance of graphene nanoplatelets reinforced calcium silicate composite. PLoS ONE. 2014;9:e106802. 10.1371/journal.pone.0106802. 25229540 10.1371/journal.pone.0106802 PMC4167702

