# Supplementary figures and images for: Fluorinated graphene-modified biodentine: an in vitro study on its ion release, cell growth, differentiation potential, and compressive strength
Source: BMC Oral Health. 2025 Oct 31;25:1716. doi: 10.1186/s12903-025-06947-7 (PMC12579429; doi:10.1186/s12903-025-06947-7)

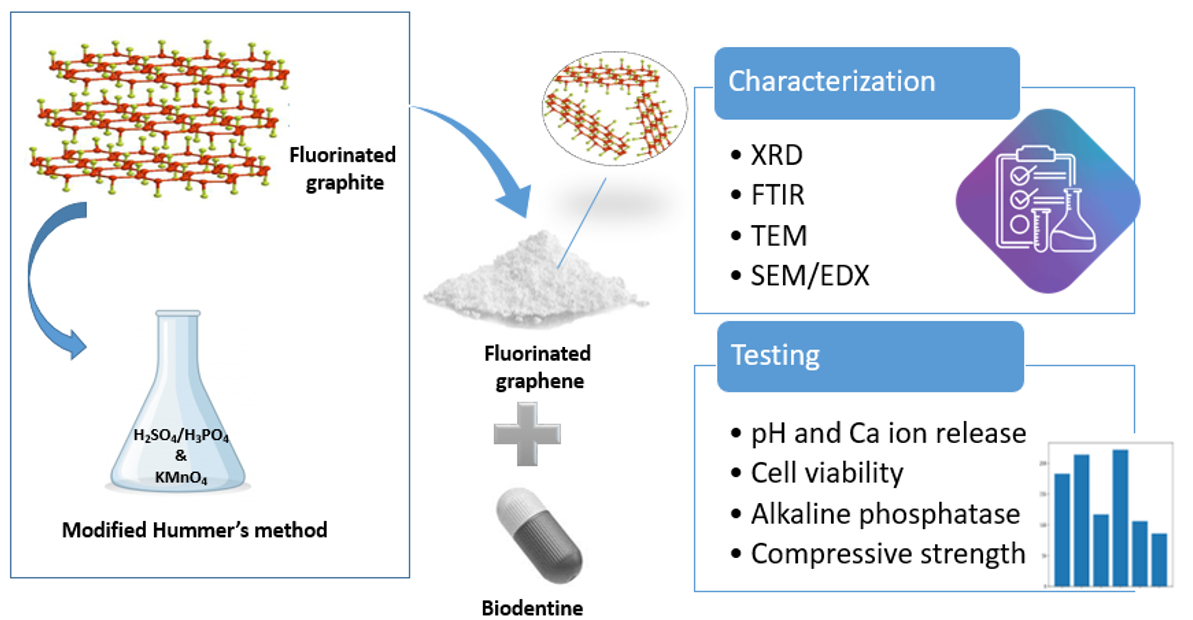

Supplement: Supplementary file 1 — Supplementary Material 1. [file 12903_2025_6947_MOESM1_ESM.tif]
